# Clinical applications of retrograde autologous priming in cardiopulmonary bypass in pediatric cardiac surgery

**DOI:** 10.1590/1414-431X20165138

**Published:** 2016-04-26

**Authors:** G.W. Fu, Y.F. Nie, Z.Y. Jiao, W.Z. Zhao

**Affiliations:** Department of Cardiovascular Surgery, The First Affiliated Hospital of Zhengzhou University, Zhengzhou, Henan, China

**Keywords:** Cardiopulmonary bypass, Retrograde autologous priming, Cardiac surgery

## Abstract

Retrograde autologous priming (RAP) has been routinely applied in cardiac pediatric cardiopulmonary bypass (CPB). However, this technique is performed in pediatric patients weighing more than 20 kg, and research about its application in pediatric patients weighing less than 20 kg is still scarce. This study explored the clinical application of RAP in CPB in pediatric patients undergoing cardiac surgery. Sixty pediatric patients scheduled for cardiac surgery were randomly divided into control and experimental groups. The experimental group was treated with CPB using RAP, while the control group was treated with conventional CPB (priming with suspended red blood cells, plasma and albumin). The hematocrit (Hct) and lactate (Lac) levels at different perioperative time-points, mechanical ventilation time, hospitalization duration, and intraoperative and postoperative blood usage were recorded. Results showed that Hct levels at 15 min after CPB beginning (T2) and at CPB end (T3), and number of intraoperative blood transfusions were significantly lower in the experimental group (P<0.05). There were no significant differences in CPB time, aortic blocking time, T2-Lac value or T3-Lac between the two groups (P>0.05). Postoperatively, there were no significant differences in Hct (2 h after surgery), mechanical ventilation time, intensive care unit time, or postoperative blood transfusion between two groups (P>0.05). RAP can effectively reduce the hemodilution when using less or not using any banked blood, while meeting the intraoperative perfusion conditions, and decreasing the perioperative blood transfusion volume in pediatric patients.

## Introduction

With the increasing number of performed cardiac surgeries, priming technique in cardiopulmonary bypass (CPB) has become an important area of research. Complex cardiovascular surgery will often require a large amount of banked blood or blood products, which are commonly limited, and may cause immune response problems, virus dissemination, and others. This encourages physicians to explore blood conservation measures that can reduce the need for allogeneic blood transfusion. At the same time, priming of conventional crystal solution in CPB will inevitably cause serious hemodilution and reduction of plasma colloid osmotic pressure, which will produce adverse effects (1-4). It has been demonstrated that the applications of retrograde autologous priming (RAP) in adult rheumatic heart disease and cardiac surgeries for coronary heart diseases can improve the hematocrit (Hct) level, reduce postoperative chest drainage volume and allogeneic blood transfusion, indicating that RAP is a safe and cost-effective blood conservation technique (5-8). The application of RAP in pediatric CPB can reduce priming volume, and keep a high Hct level during trans-instrument process (9). However, this procedure is carried out in pediatric patients with more than 20 kg, and research about its application in pediatric patients with less than 20 kg is still scarce. In infants and young children blood volume is small, and therefore the effect of RAP on hemodynamic is greater than in adults. Therefore, the application of RAP to infants and young children is limited.

Our research center has been successfully applying RAP to adults and children >20 kg. We believe that, as blood volume of infants is less than of adults, moderately reducing the volume of priming solution could result in an improved outcome in mitigating the hemodilution. In this study, we applied RAP in CPB in pediatric patients with body weight within 15 and 20 kg, and investigated whether it can reduce the perioperative blood transfusion volume.

## Subjects and Methods

### Subjects

**Figure 1:**
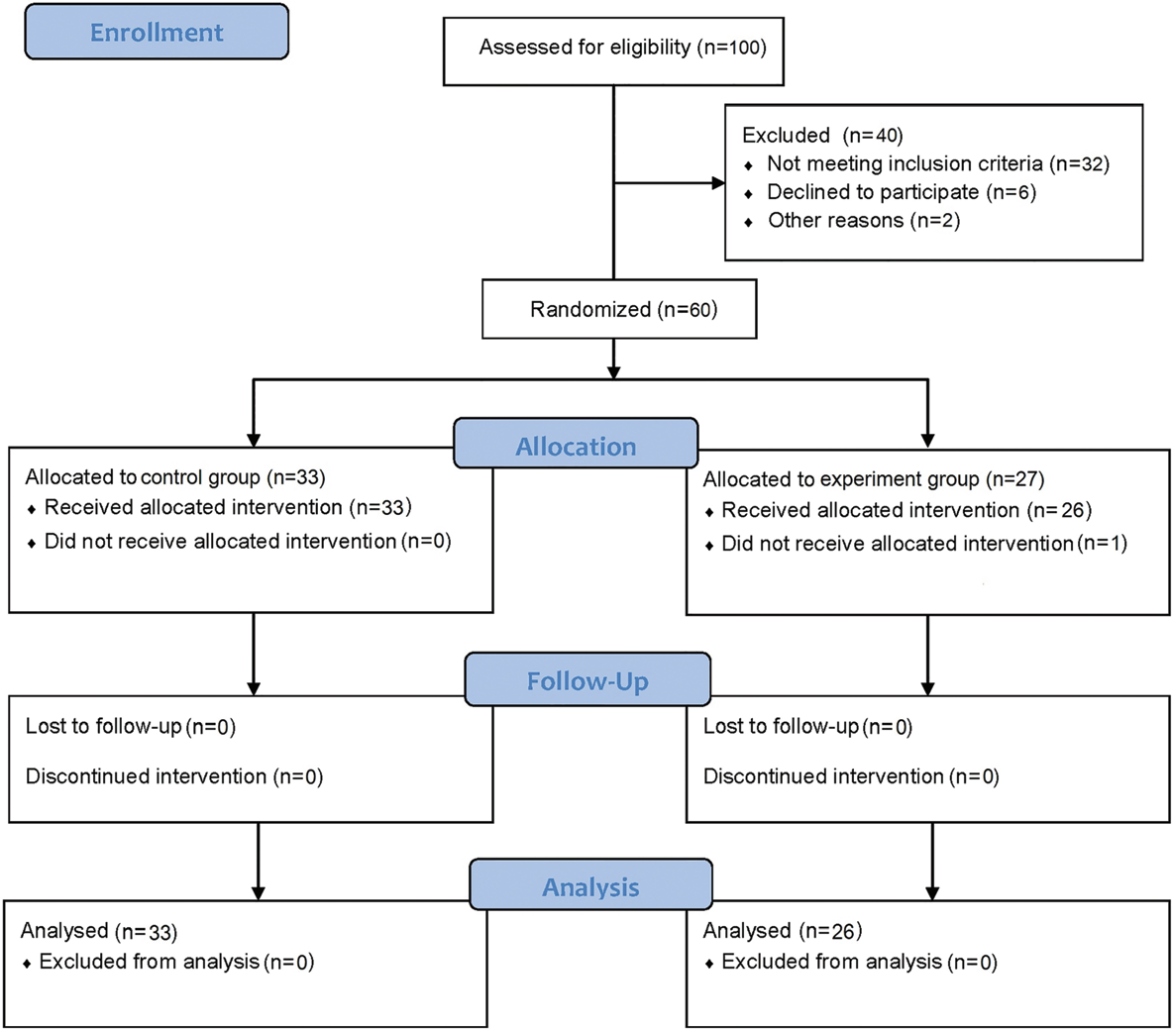
CONSORT diagram describing study flow of the participants through enrolment, allocation, follow-up and analysis phases of the trial.

This study was approved by the ethics committee of Zhengzhou University and written informed consent was obtained from the patients or their families. One hundred pediatric patients with congenital heart disease, admitted to the Department of Cardiac Surgery, First Affiliated Hospital of Zhengzhou University, who required CPB from September 2013 to June 2014, were invited to participate. Inclusion criteria were: body weight of 15-20 kg; preoperative hemoglobin (HB) level higher than, or equal to 100 g/L; and were elective for CPB intracardiac correction. Exclusion criteria were: CPB longer than 120 min and exhibited difficulties in removing treatment instruments. According to the above criteria, a total of 60 patients were included. For this single-blind experiment, the patients were divided in control group (n=33) and the experimental group (n=27) using the random number table technique. Among the control group, 12 patients were submitted to auricular septal defect repairing (ASDR), 19 patients were submitted to ventricular septal defect repairing (VSDR), and 2 patients had tetralogy of Fallot (TOF) repair. Among the experimental group, 11 patients had ASDR, 14 patients had VSDR, and 2 patients had endocardial cushion defect procedure. All patients in the control group completed the surgery, while 1 patient in the experimental group was excluded because the operation time was longer than 120 min. The CONSORT diagram is shown in Figure 1. The general information of the two groups is shown in Table 1.

**Table:**
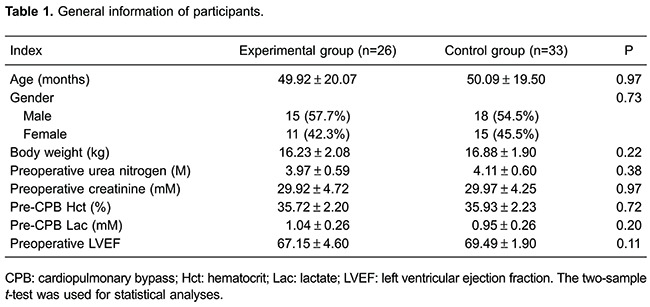

### Surgical methods

A longitudinal incision was performed at the sternum median or the fourth right subaxillary rib, and the heart was exposed. The ascending aorta and right atrium were isolated, and the F14 and F20 pipe were inserted, respectively, to connect CPB circuit (standard A type for infants; composed by 3/8×1/4 inch pipeline; total volume of 600 mL; Xijing Medical Supplies Co., Ltd., China), for establishing the CPB. The infantile membrane oxygenator (Xijing Medical Supplies Co., Ltd.) was used intraoperatively. After body temperature was cooled to 34°C, the ascending aorta was blocked, and the antegrade perfusion with 4^o^C crystal cardioplegic solution (20 mL/kg) was performed, followed by another intraoperative perfusion after 30 min interval (1/2 of the first volume). The cardiac blocking time was 20-60 min. Iced saline gauze and ice crumbs were placed on heart surface to protect myocardium. The CPB continued to operate after ascending aorta was opened, for a duration of not less than 1/4 of the aortic blocking time. After surgery, patients were monitored and treated in the intensive care unit (ICU).

### RAP method

All children were actively supplemented with crystalloid or colloid solution before surgery, to avoid lack of circulating blood volume due to fasting.

For the experimental group a sodium chloride compound priming solution was used to pre-fill the circulation pipes and exhaust air. After the patients were heparinized, the aortic cannulation was connected, and the inner loop and connecting pipe were opened, so that the blood inside the arterial pipe could slowly return and replace the same amount of priming solution (stored in a spare bag). When the pipe from the pulmonary artery membrane to the aortic cannulation site was completely filled with blood, the arterial pipe was clamped. The vena cava cannulation was connected, and the occlusion clamp of venous drainage tube was slowly opened. The venous blood was used to completely replace the liquid inside the venous tube. Meanwhile, the same amount of liquid was pumped and stored in a spare bag. During surgery, blood pressure, electrocardiogram and blood oxygen saturation of patients were closely monitored. If necessary, vasoactive agents were used to reduce the adverse effect of RAP on hemodynamics. If blood pressure dropped to <60 mmHg, 4-10 µg desoxyepinephrine was immediately injected *iv* to elevate blood pressure. If no reaction on blood pressure was achieved after desoxyepinephrine injection, the RAP was immediately interrupted, and the priming with suspended red blood cells, plasma and albumin was performed.

In the control group, the volume of banked blood was calculated as follows: Volume_banked blood_ = 600 × Hct_target_ - blood volume × (Hct_preoperative_ - Hct_target_)/Hct_banked blood_. The banked blood, 100 mL of plasma and 10 g of 25% albumin were added to CPB circuit to replace the exact volume of the priming solution.

### CPB management

The surgeries were performed using CPB machine from Stocker S5, (Italy). During CPB operation, the exogenous liquid input was reduced, and the conditions maintained as follows: colloid osmotic pressure >12 mmHg, temperature 21-23°C, average blood pressure >40 mmHg, CPB flow 120-150 mL/kg. When the average blood pressure dropped to <40 mmHg, 4-10 µg desoxyepinephrine was immediately *iv* injected to elevate blood pressure. When CPB flow dropped to <120 mL/kg, 20-30 mL of sodium chloride compound solution was *iv* injected. If the CPB flow improved, with Hct >0.25, CPB operation was continued. If CPB flow did not improve, the operation was immediately interrupted, and priming with suspended red blood cells, plasma and albumin was performed. After CPB, the ultrafiltration and transfusion of blood products were used according to the patient Hct level (target Hct >0.27) and colloid osmotic pressure.

### Observation of related indicators

Hct and lactate (Lac) levels were recorded before surgery. During surgery, the CPB time, aortic blocking time and the intraoperative blood transfusion were recorded. In addition, Hct and Lac values at 15 min after the beginning (T2) and at the end (T3) of CPB were recorded. After surgery, the mechanical ventilation time, ICU time, hospitalization duration and postoperative blood transfusion were recorded. In addition, the Hct value at 2 h after surgery (T4) was recorded.

### Statistical analysis

Statistical analysis was carried out using the SPSS17.0 software (SPSS Inc., USA). Data are reported as means±SD. Comparisons between the two groups were performed using the two-sample *t*-test. P<0.05 was considered to be statistically significant.

## Results

### Overall treatment outcome

One case in the experimental group was excluded because the operation time was longer than 120 min. All patients of the experimental group completed RAP, and only 2 patients were administrated desoxyepinephrine for unstable blood pressure. The experimental group significantly reduced priming amount, and 17 patients had no allogeneic blood transfusion perioperatively, while 26 patients of the control group received allogeneic blood transfusion. All patients were discharged successfully, and exhibited no blood transfusion-induced complications during hospitalization.

### Comparison of general information

There were no significant differences in gender, age, body weight or other general information between two groups (P>0.05). Furthermore, the preoperative Lac, creatinine, urea nitrogen, left ventricular ejection fraction (LVEF) and Hct levels between the two groups showed no significant difference (P>0.05; Table 1).

### Comparison of intraoperative indicators

There were no significant differences of CPB time, aortic blocking time, T2-Lac or T3-Lac between the two groups (P>0.05). However, the T2-Hct and T3-Hct values, and intraoperative blood transfusion exhibited significant differences between the two groups (P<0.05; Table 2). Hct levels in the experimental group were lower than those in the control group, but still maintained at >0.25 (except in two cases), which met the requirement for intraoperative blood management (Hct >0.25). In addition, the blood gas results were normal, and there was no difference in oxygen metabolism between the two groups, indicating that hemodynamics was stable during CPB in both groups. In order to further improve the Hct level (the target being >0.27), the modified ultrafiltration was performed in both groups. According to the residual blood volume in CPB circuit, the ultrafiltration volume was set as 300-450 mL.

**Table:**
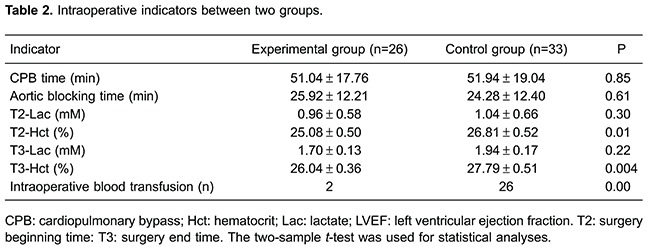

### Postoperative indicators

There was no significant difference in T4-Hct value, mechanical ventilation time, ICU time, hospitalization duration or postoperative blood transfusion between the two groups (P>0.05; Table 3). At 2h postoperative, Hct levels in experimental group were higher than the control group, but the difference was not significant.

**Table:**
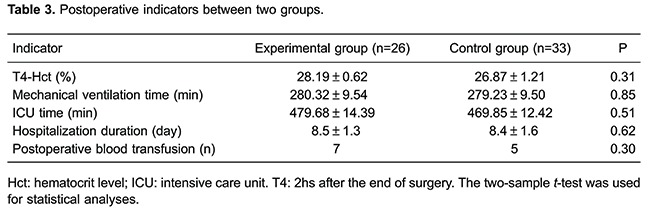

## Discussion

There exist various degrees of hemodilution in CPB, which may exhibit advantages such as reduced peripheral vascular resistance, improved microcirculation perfusion, and reduced blood destruction. Excessive hemodilution may lead to kidney damages and affect other organs' perfusion. Therefore, moderate hemodilution is an important part of CPB management (10). Blood conservation has already been vastly studied in CPB research, which includes preoperative autologous preservation, intraoperative hemodilution, and autologous transfusion (11,12). Many years of clinical trials, as well as the improvement of artificial membrane oxygenators and CPB pipelines, resulted in the progress of adult CPB, advancing from blood priming to almost bloodless priming, currently. In pediatric CPB, priming amount should be relatively larger. Therefore, the need for allogeneic blood priming still cannot be avoided in small children. However, apart from having a high risk for immune response problems and disease transmission, banked blood may have shortcomings such as decreased erythrocyte deformability, hemolysis, acidosis, abnormal inflammatory responses of white blood cells, and others (13-15). Therefore, in recent years, studies are aiming at reducing allogeneic blood priming in pediatric patients, and important progress has been achieved in children and infants with enough body weight (>20 kg).

This study targeted pediatric patients with body weight within 15-20 kg. Our results indicated that the experimental group, which did not use banked blood, obtained outcomes similar to the control group. Patients with body weight <20 kg have less blood volume then necessary for RAP, which would likely affect hemodynamic stability. Therefore, to perform RAP, blood volume should be positively supplemented before surgery, thus avoiding inadequate circulating blood volume, caused by fasting. During this operation, the patient’s blood pressure, echocardiogram and oxygen saturation should be closely monitored, and anesthesiologists, surgeons and CPB physicians should cooperate closely. Vasoactive drugs should be administered when necessary to reduce the adverse effects of RAP towards hemodynamics. As for patients who show poor heart functions, or signs of intolerance for the RAP technique, the operation should be interrupted promptly. Furthermore, this technique must consider the overall condition of the patients, and a combination with other blood conservation methods, such as modified ultrafiltration, might be considered to achieve the best blood-protective effects and improve prognosis (16,17). Successful implementation of the combined application of RAP and other blood-saving methods has been reported (18,19).

In cardiac surgery, the probability of using allogeneic blood in infants and young children is relatively higher than in adults. At present, blood source is relatively limited, therefore using less or not using any banked blood can be an advantage. The successful application of RAP in children with body weight <20 kg can result in satisfactory Hct levels in CPB, and maintain stable hemodynamics. This can effectively ease the situation of lack of banked blood, and avoid the risk of various complications and infectious diseases related to blood transfusion. In conclusion, RAP can effectively reduce the hemodilution in CPB when using less or not using any banked blood, while meeting the intraoperative perfusion conditions, and decreasing the perioperative blood transfusion volume in pediatric patients.
